# Radiation source detection for the accurate location of lymph node metastases using robotic forceps-type coincidence radiation detector

**DOI:** 10.1007/s11548-024-03296-8

**Published:** 2024-12-02

**Authors:** Kazuya Kawamura, Ayano Nakajima, Shigeki Ito, Miwako Takahashi, Taiga Yamaya

**Affiliations:** 1 Center for Frontier Medical Engineering, Chiba University, Chiba, Japan; 2 Graduate School of Science and Engineering, Chiba University, Chiba, Japan; 3 Mirai-Imaging Corporation, Fukushima, Japan; 4 National Institutes for Quantum Science and Technology, Chiba, Japan

**Keywords:** Coincidence radiation detecting, Determination of point-spread function, Lymph node dissection, Esophageal cancer

## Abstract

**Purpose:**

We have developed a forceps-type coincidence radiation detector for supporting lymph node dissection in esophageal cancer treatment. For precise detecting, this study aims to measure the 2D point-spread function of the detector at three difference tip angles, to devise a method to determine the position of a point source using the 2D point-spread function.

**Method:**

The 2D sensitivity distribution on the surface of the detector was investigated to assess sensitivity variation caused by differences in the relative positions of the detector and radiation source. Based on the results, we identified the peak sensitivity value and proposed a detection method using this value. We evaluated the effectiveness of the proposed method by detecting radiation source location using this simulated distribution.

**Result:**

From the radiation sensitivity distribution measurements, we observed a gradual decrease in radiation detection sensitivity from the center toward the edges of the detector surface. Additionally, we verified that the peak sensitivity value was attainable. Through the basic verification of the detection method, we confirmed that the radiation source location could be detected within a maximum error of 1.4 mm.

**Conclusion:**

We developed a peak value search method aimed at mitigating sensitivity variations by leveraging the sensitivity distribution across the detector surface. The proposed device is thought to be able to quantitatively evaluate the desired target assuming that the field of view could be limited to the area clamped by the detector. As a next research step, more precise search methods should be verified in an environment resembling the one of the target clinical uses.

## Introduction

### Background

Three-field lymph node dissection is a standard procedure for the radical treatment of esophageal cancer. However, the increasing incidence of postoperative complications can be attributed to its high invasiveness [1, 2]. Minimally invasive procedures have shown efficacy in mitigating these postoperative complications. Recently, surgical robotics and specular surgery have gained popularity as less invasive alternatives for treating esophageal cancer [3, 4]. Notably, surgical robotics have demonstrated significant suppression in postoperative complications compared to open surgery [5]. Furthermore, reducing the extent of resection has been found to effectively decrease postoperative complications [2]. Yoshimura et al. showed that unnecessary resections happen if metastatic state is not considered when deciding extension of lymph node dissection in esophageal cancer [6]. Extensive resection is often unavoidable due to challenges in intraoperatively identifying the exact locations of metastases [1]. Accurately determining the scope of lymph node resection is crucial for reducing postoperative complications by minimizing unnecessary resections and avoiding resection in high-risk areas.

Preoperative imaging techniques, including computed tomography (CT) and positron emission tomography (PET), play a crucial role in diagnosing preoperative lymph node metastases in patients with esophageal cancer [6]. PET is valued for its ability to detect early cancerous changes, as biochemical changes in tumors often precede morphological ones. The report by van Vliet et al. highlighted the diagnostic advantage of PET, particularly with the use of 18F-fluorodeoxyglucose (FDG-PET), over CT in identifying cancerous lesions [7]. However, it also drew attention to a significant limitation: The sensitivity of FDG-PET in diagnosing regional lymph node metastases might be compromised by the PET scanner’s limited spatial resolution. Although the synergistic use of CT and FDG-PET has been investigated for preoperative lymph node metastases, the challenge of spatial resolution persists. Additionally, the simultaneous use during the surgical procedure remains unachievable because intraoperative measuring the condition of the affected area using CT or FDG-PET is difficult [6]. The dynamic nature of intraoperative organ movement and patient positioning complicates the precise correlation of metastatic sites identified in preoperative imaging with their actual intraoperative locations. Therefore, improving the spatial resolution of diagnostic imaging equipment and translating preoperative imaging data into actionable intraoperative information are crucial steps toward accurately identifying all metastatic sites based on preoperative lymph node metastasis diagnosis.

### Related works

Intraoperative molecular imaging and radiation-guided navigation surgery using 18F-FDG as a biomarker have been investigated as methods for precisely targeting metastatic sites during surgery [6, 8–10]. Nwogu et al. showed that metastases undetectable in preoperative PET scans could be identified intraoperatively with the aid of a portable gamma probe [11]. This result suggests that the specificity of 18F-FDG, coupled with measurements taken in proximity to the lymph nodes, can achieve a higher spatial resolution than preoperative PET. Furthermore, Yoshimura et al. investigated the efficacy of using18F-FDG in the intraoperative detection of lymph node metastases in esophageal cancer, suggesting that 18F-FDG could serve as a useful intraoperative biomarker [6]. For diagnostic and therapeutic oncology where the target tissue is surrounded by organs with high uptake in PET, a multimodal instrument, such as combination of PET with another modality, has been proposed [12]. For navigation surgery using 18F-FDG as a biomarker, several intraoperative radiological measurement techniques have been developed [11, 13–15]. These methods use either a handheld gamma probe detector or a DROP-IN gamma probe detector, which is placed inside the body and maneuvered with forceps for scanning. These results suggest potential solutions to challenges in preoperative diagnosis, such as the need for expedited diagnosis using medical imaging and enhancing the spatial resolution of diagnostic imaging system. However, limitations persist in the spatial resolution of portable gamma probes, and the interference from background radiation—stemming from physiological uptake, respiration, and lymph node movement associated with cardiac activity—underscores the need for a new imaging device [6].

### Purpose

18F-FDG has demonstrated utility as an intraoperative biomarker in esophageal cancer. However, portable gamma probes used for intraoperative radiological measurements encounter some problems, including radiation contamination from physiological accumulation sites and difficulties in distinguishing FDG activity signals in the lymph nodes surrounding the primary tumor. Currently, single radiation measurements using handheld probes are in practical use, where gamma rays from the desired direction can be detected using a single detector and collimator. However, due to changes in sensitivity with distance and the contribution of radiation from sources other than the target, it is not possible to obtain quantitative values. Therefore, this method is limited in its use for ambiguous location estimation, such as intraoperative detection of sentinel lymph nodes. In recent years, research has been conducted on the use of beta rays and imaging using external pixel-type detectors. Beta rays are absorbed in tissues; therefore, a solution for false negatives is needed. For imaging using external detectors, technical improvements are required to obtain practical images within a realistic measurement time that can be secured during surgery. In a detailed review and position paper, Fragoso et al. proposed and identified the possibilities and challenges that relate to the successful implementation of β-emitters in surgical guidance, covering aspects related to instrumentation, radiation protection, and modes of implementation [16].

**Fig. 1:**
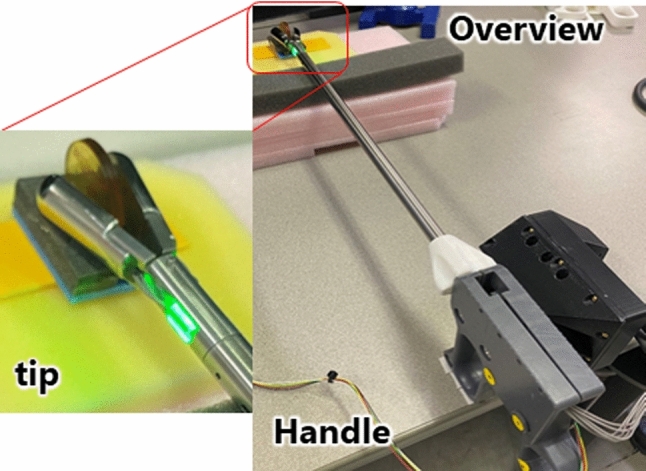
Forceps-type coincidence radiation detector

To address these challenges, we have been proposed a sensing device for an FDG-guided surgery targeting high metabolic uptake lymph nodes. Takahashi et al. proposed a forceps-type coincidence radiation detector to incorporate the simultaneous intraoperative radiation measurement technology of PET [17]. The concept of coincidence radiation counting was first reported in 1997[18]. However, the parts for radiation measurement were too big, and the device could be miniaturized at that time. In this device, a pair of detectors from a clinically used PET scanner are miniaturized and mounted on the tip of the forceps (Fig. 1). The proposed device is thought to be able to quantitatively evaluate the desired target because the field of view could be limited to the area clamped by the detector. The detector was developed and evaluated in our previous study [19]. This configuration allows for the detectors to be positioned at the tip of the forceps, enabling coincidence radiation detecting by pinching suspected metastases during the operation.

**Fig. 2:**
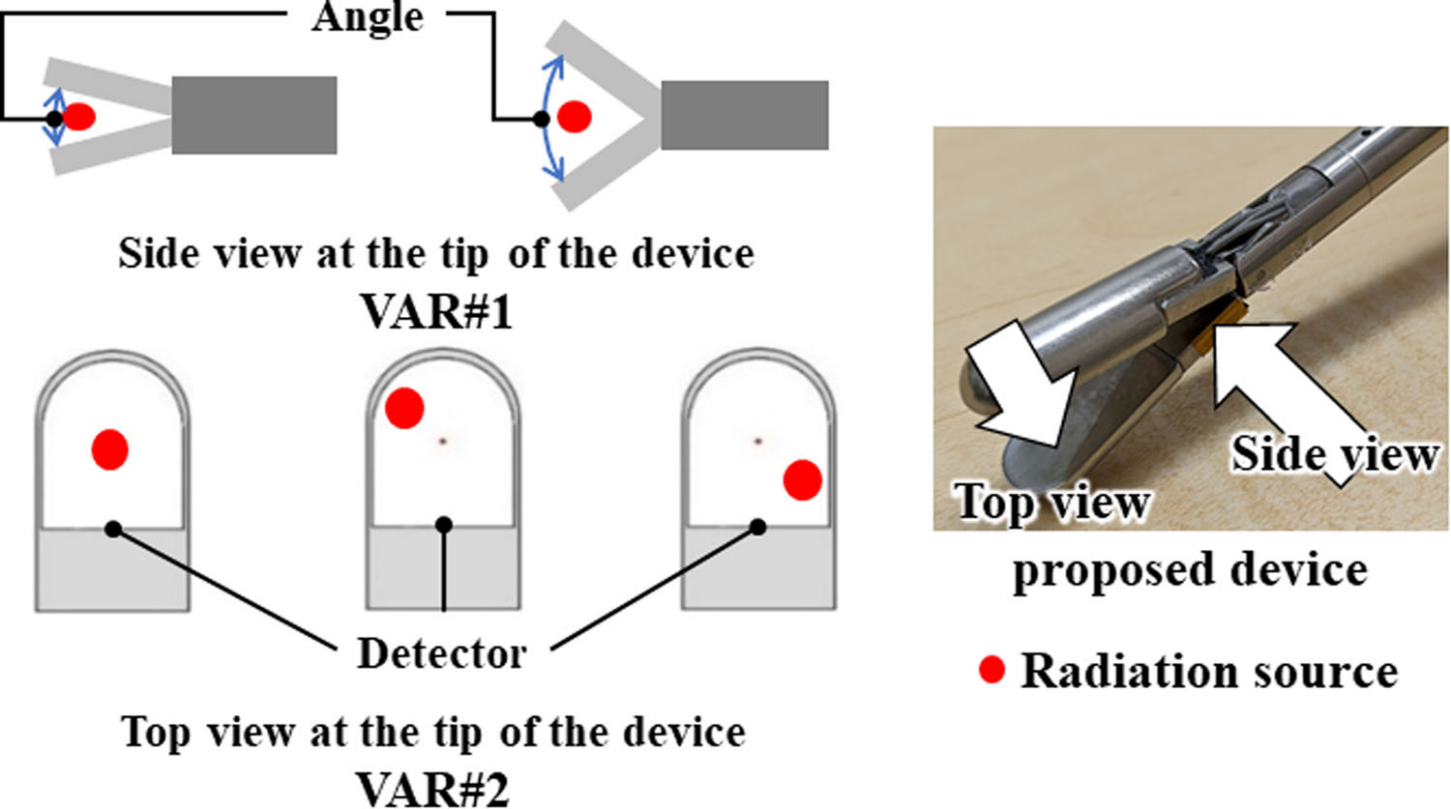
Variation factor of forceps-type coincidence radiation detector

However, two issues concerning the sensitivity of radiation detection arise when using this measurement method. The first issue is sensitivity variation stemming from differences in the angle between the detector and radiation source (VAR#1), while the second issue is sensitivity variation arising from differences in the relative positions of the detector and radiation source (VAR#2) (Fig. 2). VAR#1 arises due to the challenge of maintaining consistent radiation detection sensitivity, as the angle between the detector and the radiation source constantly change. Because the device is operated manually by the surgeon, these changes in angle are influenced by the position of the device’s tip when clamped to the patient’s body and by the size variations of the target object.

In a previous study, Nakajima et al. developed a sensitivity correction method [20] to reduce and improve the VAR#1, assuming that VAR#2 did not occur. Their focus was on the correlation between the tip angle of the forceps and the distance between a pair of detectors. Through their work, they demonstrated that sensitivity variation could be improved by deriving a correction formula from the relationship between the tip angle of the forceps and the radiation count rate in forceps-type coincidence radiation detector.

In this study, we focus on resolving VAR#2. During a preliminary investigation, we measured the sensitivity distribution of radiation detection across the detector surface. Our observations revealed two notable trends: firstly, an exponential decrease in radiation detection sensitivity, and secondly, a general decline in the radiation count rate toward the edges of the detector surface. Additionally, we observed the presence of a peak sensitivity in radiation detection. This phenomenon is likely attributable to the broader three-dimensional angle within which radiation can be detected in the central portion of the detector, and the longer intracrystal distance through which radiation can traverse. From the preliminary investigation, we thought that if the detector characteristics could be utilized to perform radiation measurements in a state where the maximum sensitivity point of the detector aligns with the radiation source position, it could mitigate sensitivity variations due to the differences in the relative positions of the detector and radiation source (VAR#2). Therefore, the purpose of this study is to develop a peak value detection method to improve sensitivity variation. This method will be based on the detection sensitivity distribution across a finely divided detector surface.

## Experiment

### Development of peak value detection method

**Fig. 3:**
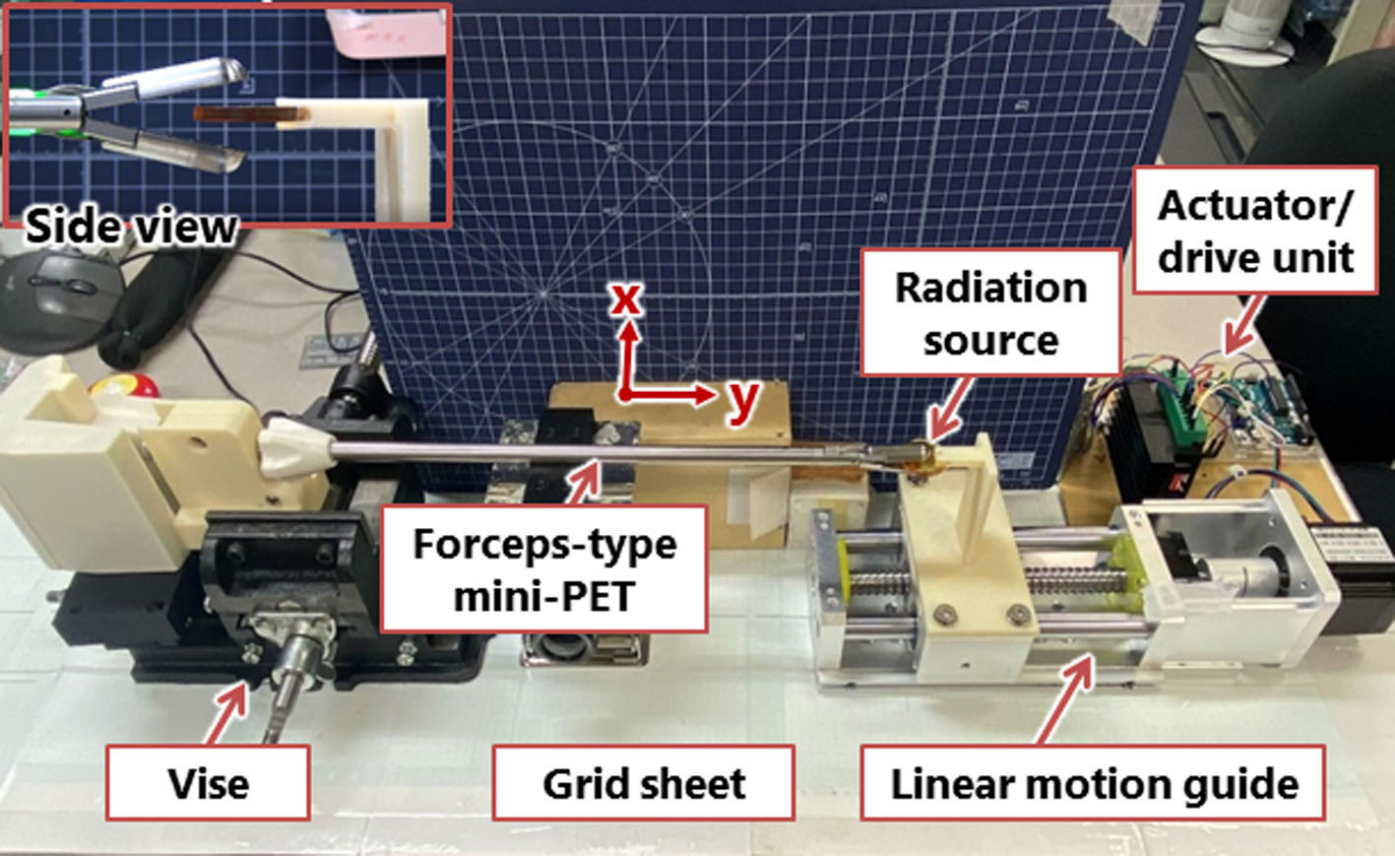
Experimental setup for measurement of sensitivity distribution

In this experiment, we investigated the sensitivity distribution of the forceps-type coincidence radiation detector to verify the sensitivity variation resulting from differences in the relative positions of the detector and radiation source. The diameter of the radiation source (^22^Na point source) was set at 1 mm (RADIOACTIVE Na-22 HY965, QSA Global Inc., MA, USA). Given this configuration, achieving peak sensitivity in radiation detection required a resolution of at least 1 mm to identify the peak radiation dose. Therefore, we obtained and verified the sensitivity distribution of radiation detection on the detector surface through 1 mm resolution plots. The proposed device was developed as an instrument that can pinch the radiation source at any position on the detector surface at the tip of the device. Because it is not possible to identify where the source is located on the detector surface, it is not possible to refer to a lookup table, which is a common method. Therefore, we searched for the most sensitive part of the detector by measuring the variation in the coincidence counting. The experimental setup is illustrated in Fig. 3. The radiation dose was measured by varying the position of the point source on the detector surface. The body of the forceps-type coincidence radiation detector was secured to a vise (CR75N; TRUSCO, Tokyo, Japan). Both the device and the linear motion guide equipped with a stepping motor (B07V48B7X1, Heechoo, Guangdong, China), along with the radiation point source, were placed on a grid sheet. The radiation source was manually moved in the x-axis direction along the grid sheet’s scale. Furthermore, movement along the y-axis was achieved using a linear motion guide.

**Fig. 4:**
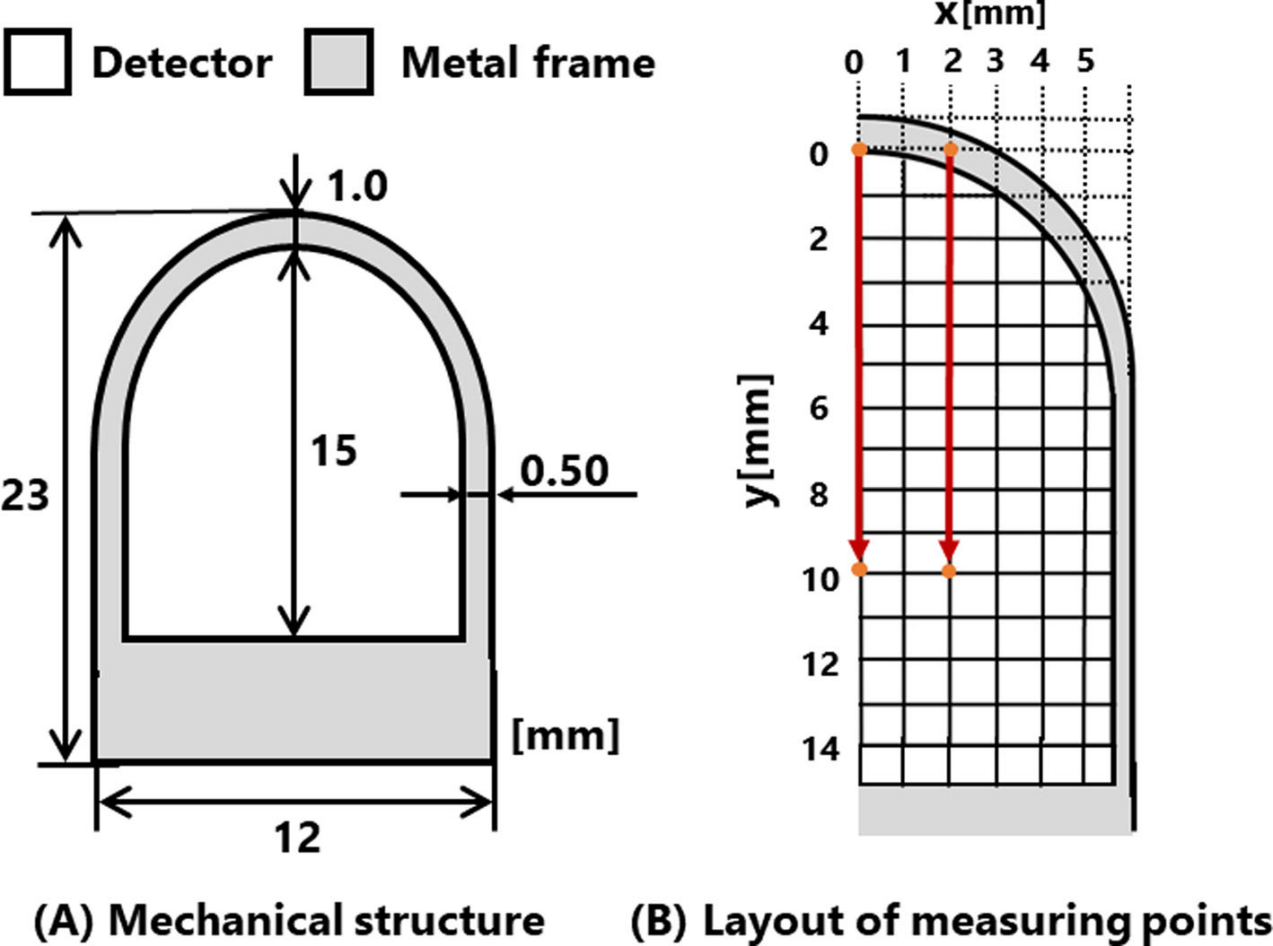
Mechanical structure of tip of forceps-type coincidence radiation detector

The radiation dose on the detector surface was obtained by maintaining a constant tip angle of the forceps and moving the point radiation source. The mechanical structure of the detector is shown in Fig. 4A. The detector surface exhibited symmetrical from the center, and measurements were taken from one half of the detector. Since the sensitivity of radiation detection depends on the angle and distance between the detector and radiation source, three different forceps tip angles (large, medium, and small) were used to investigate how these factors influence the sensitivity distribution. The angles selected were 13°, representing the minimum angle at which the forceps were closed, 22°, corresponding to the maximum angle at which the forceps were opened, and 17°, serving as an intermediate value between these angles. For measuring the forceps tip angle, a forceps tip angle measurement system equipped with a wire-type linear encoder (MLS-12, MICROTECH LABORATORY Inc., Kanagawa, Japan), developed in a previous study, was used [21]. This device has a mechanism that opens and closes the tip by pushing and pulling a rod. Therefore, the tip angle is determined based on the amount of rod traction. The amount of rod traction was measured using a linear encoder connected to the rod, and the tip angle was calculated using the amount of rod traction. The sensitivity was calculated as a percentage of the maximum number of coincidences radiation counting under each experimental condition (tip angle). This normalization ensures that the peak sensitivity for each condition is represented as 100%.

As shown in Fig. 4B, the measurement sequence was shifted by 1 mm in the y-axis direction sequentially from the coordinates of the center row (0,0) until (0,10) was measured. Similarly, measurements were performed from the top by shifting 1 mm in the x-axis direction. The measurement range in the y-axis direction spanned from 0–10 mm, while the measurement range extended from 0–11 mm only when the tip angle of forceps was 22°. The measurement range in the y-axis direction was determined to ensure that the forceps and radiation source did not make contact at each angle. Specifically, the measurement range in the x-axis direction was 0–4 mm. Any ranges exceeding 4 mm were excluded from the measurement range due to numerous points where the source was situated outside the detector. At each location, measurements were performed three times, and the average value was computed to represent the radiation dose at that point. In this experiment, the coincidence time window was set to 125 ns.

### Basic verification of proposed method in simulated environment

The radiation dose varies depending on the relative position between the radiation source and the detector. To perform a basic verification of radiation source detection using peak value searching, we must consider the variations. Therefore, in this experiment, we performed basic verification using the following steps: First, we measured the radiation dose emitted from a specific type of point source. Second, we generated verification data by introducing random noise to the measured data. Third, we calculated the simulated distribution of radiation sensitivity. Finally, we evaluated the efficacy of the proposed method by detecting radiation source locations using the simulated distribution.

**Fig. 5:**
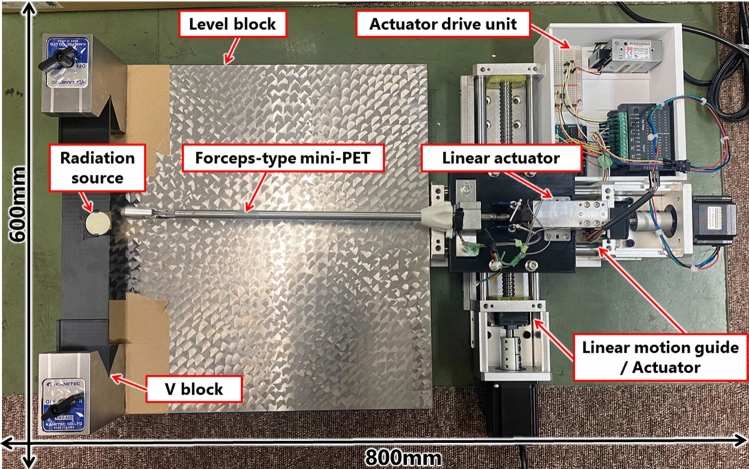
Experimental setup for verification of proposed method

**Fig. 6:**
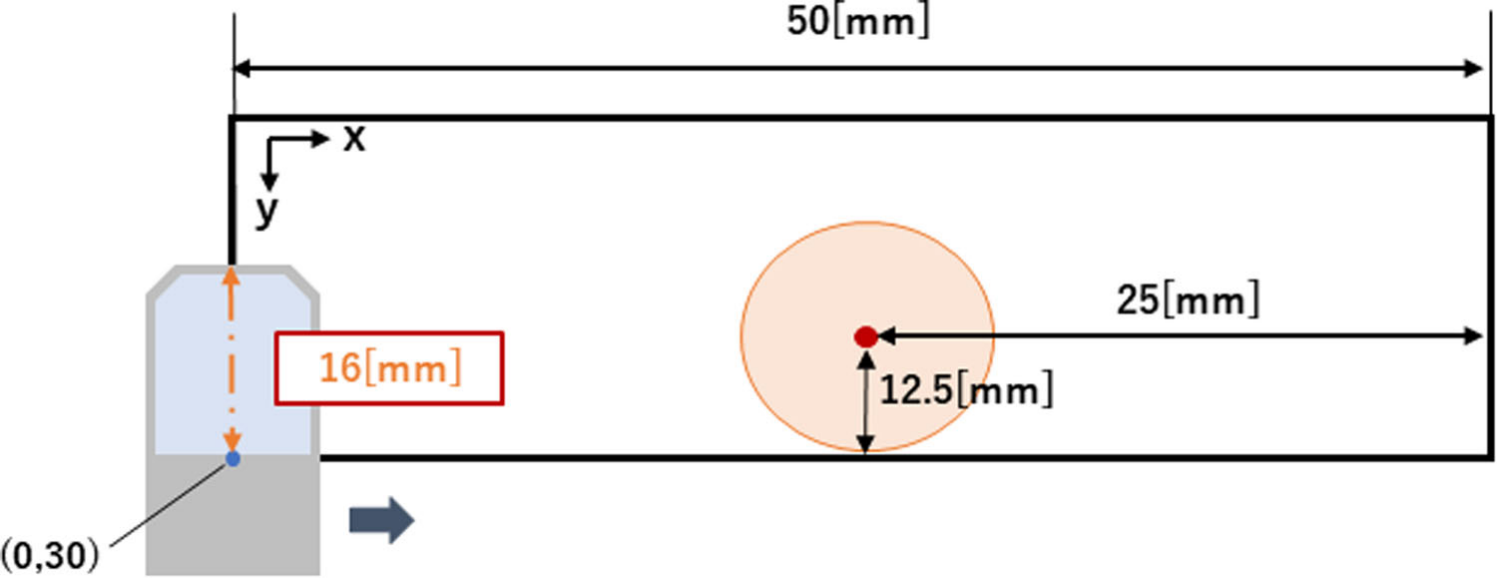
Experimental layout for detecting radiation source

The experimental setup is illustrated in Figs. 5 and 6. A sensitivity distribution of 10 kBq was obtained for the point source emitting 10 kBq. To obtain the simulated distribution, 56 points were selected near the point source on the plate where it was positioned. Measurements were performed three times at each measurement point, and the average value was calculated. Random noise was introduced within the range of the maximum and minimum radiation intensity values from the three measurements. The theoretical position of the source was determined as the maximum position of the radiation intensity in the distribution before the addition of random noise, and it was compared with the search results.

We conducted a search for the peak radiation dose value and identified the location of the radiation source through data transfer. This process simulated scanning along the x- and y-axis directions. Given that the radiation source was assumed to be a point source with a diameter of 1 mm, the search interval was set to 0.5 mm. During the search procedure, we first scanned along the x-axis direction, then moved to the detected peak value, and subsequently scanned along the y-axis direction to detect the peak value. As several peak values may appear around the radiation source, the highest peak value was selected as the search result. Subsequently, following the search, the radiation dose measured at the intersection of the peak values in the x- and y-axes was corrected using the tip angle of the forceps-type coincidence radiation detector. To implement this methodology, we employed the correction formula for radiation detection sensitivity derived from a previous study [20].

The target values for this experiment were set within ± 1.5 mm in the x- and y-axis directions from the theoretical position, assuming a sensitivity range of at least 70% relevant to the results obtained in the previous section. The theoretical value for the corrected radiation dose was 10 kBq, with the error tolerance set at ± 30% based on the characteristics of the forceps-type coincidence radiation detector as reported in previous studies.

## Results

### Development of peak value detection method

**Fig. 7:**
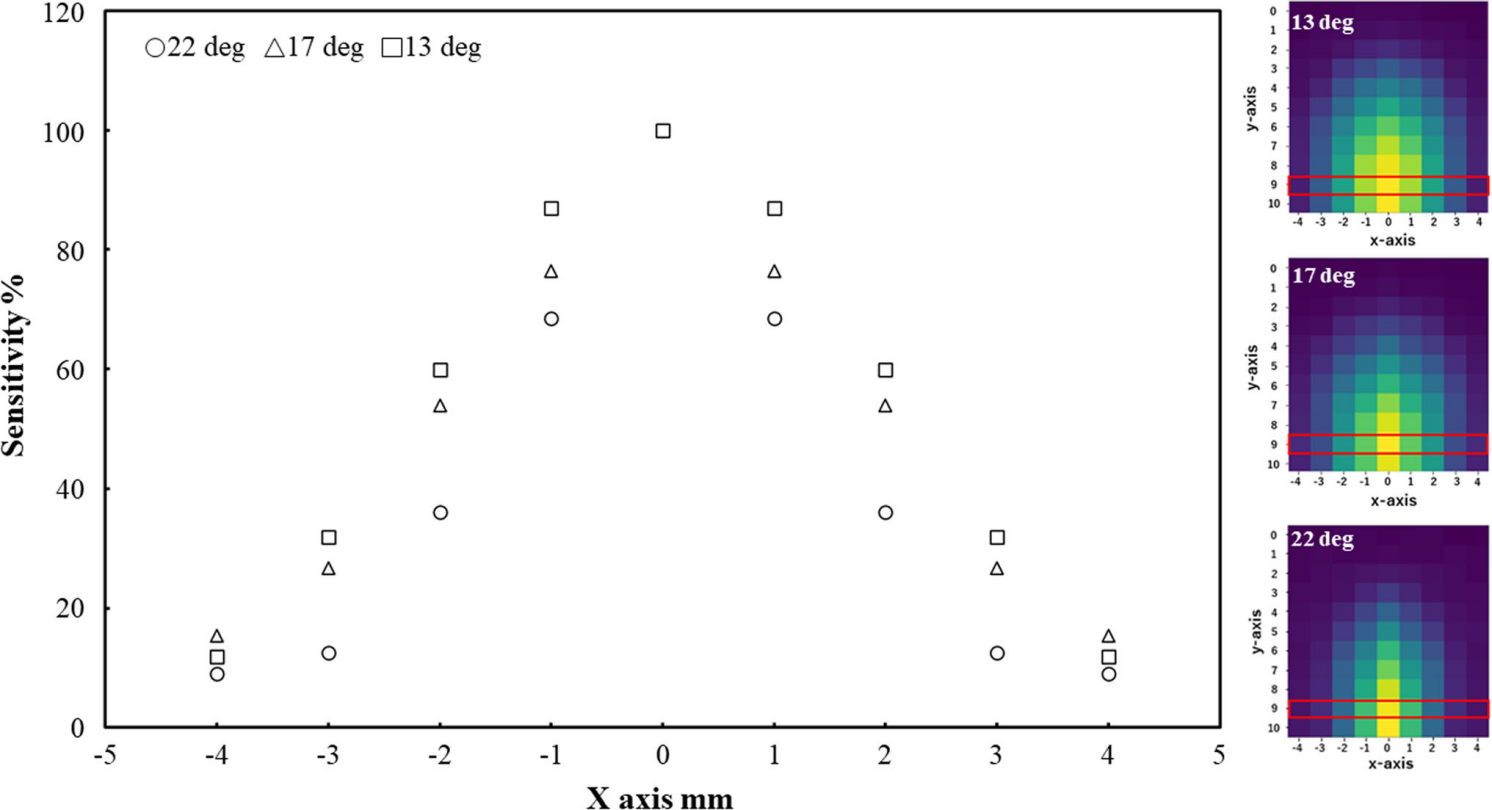
Radiation detection sensitivity in x-axis direction

**Fig. 8:**
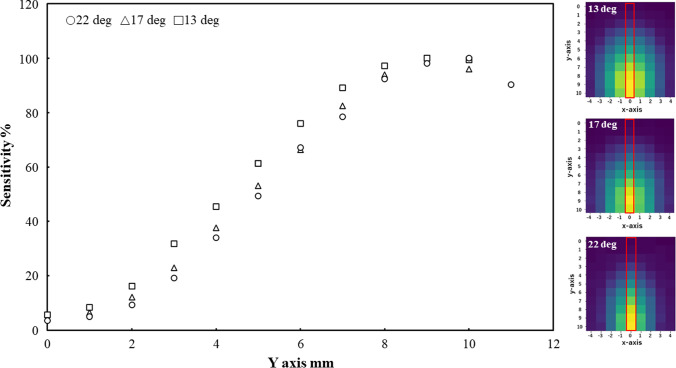
Radiation detection sensitivity in y-axis direction

Figures 7 and 8 present the radiation detection sensitivity trends and distribution results along the x- and y-axes, respectively, for each tip angle condition. In both figures, the sensitivity is shown as a scatter plot, while the distribution is represented as a heatmap. For the scatter plots, the horizontal axis shows the position along each respective axis (x or y), while the vertical axis represents the radiation detection sensitivity. In the heatmaps, the horizontal and vertical axes correspond to the x- and y-axes, respectively. The position of the maximum sensitivity point along each axis is indicated by a red box. The results were normalized based on the maximum radiation intensity obtained for each angle, with yellow indicating maximum sensitivity in the heatmaps. In the scatter plots, the values were obtained by normalizing sensitivity with respect to the maximum value of sensitivity in each direction. From Figs. 7 and 8, we confirmed a gradual decrease in radiation detection sensitivity from the center toward the edges of the detector surface. Furthermore, we also confirmed that peak radiation sensitivity occurs along both the x- and y-axes during detector scanning with a resolution of 1 mm regardless of the tip angle of the detector. This result confirms that it is possible to determine the point of maximum detection sensitivity on the detector surface from a point radiation source using the method employed in this experiment.

### Basic verification of proposed method in simulated environment

Table 1 lists the radiation intensities at the searched positions, along with the corrected radiation intensities, mean values, and standard deviations in the searched source positions obtained from 50 distributions generated after the introduction of random noise. The corrected radiation intensities and searched source positions were compared with the reference values. The results detailed in Table 1 affirm the precise correction of radiation intensity. Moreover, it was established that the location of the radiation source could be detected with maximum absolute errors of 1.1 and 1.4 mm along the x- and y-axes, respectively. Next, we calculated the radiation intensity from the experimental results using the methods of previous studies [20, 21].

**Table 1:** Search and correction results

Evaluation item	Results	References	Error
Intensity of radiation(corrected) kBq	10 ± 0.6	10	–0.0
Radiation source location (x) mm	24.5 ± 0.6	25	–0.5
Radiation source location (y) mm	20.8 ± 0.7	21.5	–0.7

## Discussions

As shown in Figs. 7 and 8, it was observed that the low-sensitivity area expanded with an increase in the forceps tip angle. Notably, the occurrence of radiation detection sensitivity peaks exhibited a consistent trend despite variations in the angle. This phenomenon led us to consider factors such as the decrease in solid angle within the detector capable of detecting radiation at its periphery, along with the diminished distance within the detector traversable by radiation. The search error of the x- and y-coordinates was within the allowable error range for source search position (within ± 1.5 mm). Based on these results, the proposed method proves effective in detecting the radiation source’s location. The mechanical shape of the detector is symmetrical along the y-axis (Fig. 4). Therefore, we thought that the sensitivity distribution in the x-axis direction could be measured symmetrically, and the maximum sensitivity was expected to measure at X = 0 since the detectable range (length of the device in the longitudinal direction) was the largest at X = 0. On the other hand, the shape is not symmetrical along the x-axis. Since the detector has both a planar and a curved ends, the sensitivity on the curved side decreases smoothly, while the sensitivity on the planar side decreases abruptly. Therefore, we thought that it is possible to estimate the peak value from this experiment. However, in this measurement in the y-axis direction, due to the size of the radiation source and the effect of the measurement variation, it is possible that the sensitivity gradually increases from the curved surface side, and the peak value may not be measured. Therefore, we thought that a higher resolution may be required to detect the peak value.

Based on these results, the proposed method proves effective in detecting the radiation source’s location. However, considering the variation in the y-axis direction, the values are barely within the acceptable error range for searching the position of the radiation point source when errors on the negative side in the y-axis direction occur. We suggested the potential solution to address radiation detection sensitivity in forceps-type coincidence radiation detector by implementing source position detection using a peak value search and a correction method using the forceps tip angle, leveraging approaches from previous studies [20, 21].

In this experiment, we used radiation maps with random noise generated using the range of the variation in coincidence counting from the point source that was the target of the measurement. The results suggest that the uncertain location of the radiation source can be identified by searching for the point with the maximum detection sensitivity at the detector in this specific environment. Combining this result with the sensitivity correction according to the angle of the detector tip, we suggest that it is possible to estimate the source intensity from the measured coincidence counts, although the true value of the point source intensity needs to be known.

For our proposed device, the ionizing dose exposure for surgical staff is a point of concern. Comparable investigations into FDG-guided surgeries utilizing probe-type detectors have reported varying results. Piert et al. reported that absorbed radiation doses ranged from 2.5 to 8.6 μSV/h when surgery was conducted 3 h after FDG administration (47.0 and 41.4 MBq) [22]. Povoski et al. reported 164 μSV when surgery commenced 142 min following FDG injection (mean dose of 699.3 MBq) [23]. Gollub et al. reported an ex vivo FDG-PET study, where surgery began 45 min after administering FDG (555–740 MBq), noted exposure doses for surgical staff between 0.4 and 0.8 mSV per procedure [24]. In our previous research, Takahashi et al. evaluated radiation exposure levels for various operating room staffs during surgical procedures. The surgeon received the highest dose per operation at 44 μSV. The variability in exposure doses likely stems from differences in injection doses and the time interval between administration and the onset of surgery. The International Commission on Radiological Protection has established an annual exposure dose limit of 50,000 μSV for healthcare professionals. Given these guidelines and our findings, we conclude that the procedure can be performed safely within the prescribed occupational radiation exposure limits.

In this study, we conducted only a search for a point radiation source on a two-dimensional plane, and the experimental environment did not consider the device going into the body via trocars assuming endoscopic surgery. For clinical application, it is necessary to verify issues such as 3D motion, size and shape of radiation sources (such as lymph node), radioactive uptake in other structures, soft tissue deformation, and breathing/heart beat motion. Moreover, achieving precise position control of the detector is imperative for using a peak value search to locate the radiation source, as opposed to relying on conventional surgeon-dependent scanning methods such as forceps-type coincidence radiation detector. Therefore, the challenges posed by environmental factors and positional errors arising from the introduction of robots or other precision-enhancing devices necessitate the exploration of even more accurate search methodologies assuming clinical environments.

## Conclusion

In this study, the variation in radiation sensitivity owing to the differences in the relative position of the detector and radiation source was studied. This issue is particularly relevant to the forceps-type coincidence radiation detector proposed as a means to reduce the dissection area in a treatment such as lymph node dissection in esophageal cancer. We developed a peak value search method for ameliorating sensitivity discrepancies, leveraging the sensitivity distribution across finely divided detector surfaces. The results from the radiation detection sensitivity distribution, analyzed at 1 mm resolution across the detector surface, show that sensitivity to radiation detection diminishes gradually from the center toward the edges of the detector surface. Moreover, the study identified the radiation detection sensitivity peaks along the longitudinal and orthogonal axial directions on the detector surface. These observations imply that by aligning the detector’s point of maximum sensitivity precisely with the position of the radiation source, it is possible to reduce sensitivity variations caused by differing relative positions between the detector and source. Therefore, validation data were generated using a point source, and the effectiveness of the search method using peak values was verified. Consequently, it was confirmed that the peak radiation dose at the measurement target could be detected. The subsequent phase of this research will perform the verification of the proposed method in more complex environment in preparation for clinical use.
